# Cancer cachexia: lessons from *Drosophila*

**DOI:** 10.1242/dmm.049298

**Published:** 2022-03-23

**Authors:** Ying Liu, Pedro Saavedra, Norbert Perrimon

**Affiliations:** 1 Department of Genetics, Harvard Medical School, Boston, MA 02115, USA; 2 Howard Hughes Medical Institute, Boston, MA 02115, USA

**Keywords:** Cachectic factors, Cancer cachexia, *Drosophila*, Organ wasting

## Abstract

Cachexia, a wasting syndrome that is often associated with cancer, is one of the primary causes of death in cancer patients. Cancer cachexia occurs largely due to systemic metabolic alterations stimulated by tumors. Despite the prevalence of cachexia, our understanding of how tumors interact with host tissues and how they affect metabolism is limited. Among the challenges of studying tumor–host tissue crosstalk are the complexity of cancer itself and our insufficient knowledge of the factors that tumors release into the blood. *Drosophila* is emerging as a powerful model in which to identify tumor-derived factors that influence systemic metabolism and tissue wasting. Strikingly, studies that are characterizing factors derived from different fly tumor cachexia models are identifying both common and distinct cachectic molecules, suggesting that cachexia is more than one disease and that fly models can help identify these differences. Here, we review what has been learned from studies of tumor-induced organ wasting in *Drosophila* and discuss the open questions.

## Abstract (precis)

**Summary:** *Drosophila* tumor models have contributed to our knowledge of cancer cachexia. Here, we review current knowledge on *Drosophila* cachectic factors, their effects on host organs and metabolism, and discuss open questions for future studies.

## Introduction

Cachexia is a general state of weight and muscle loss due to illness. It frequently occurs in cancer patients, especially at the more advanced stages of the disease (Dewys et al., 1980; Tisdale, 2009). Cancer cachexia is characterized by a marked loss of body weight, as well as by anorexia, asthenia and insulin resistance (see Glossary, Box 1) (Argilés et al., 2007; Dhanapal et al., 2011; Giordano and Jatoi, 2005), and has been estimated to cause 20-40% of cancer patient deaths (Bruera, 1997; Tisdale, 2009). Although no universal physiological mechanism of cancer cachexia has been identified, it is generally agreed that it is caused by a metabolic imbalance triggered by tumors that perturbs physiological homeostasis.

**Box 1. Glossary**

**Adipose triglyceride lipase**: a lipase that catalyzes the hydrolyzation of triacylglycerols to diacylglycerols – the first step in lipolysis.

**Anabolic metabolism**: also termed anabolism; a group of metabolic pathways that constructs molecules from smaller units.

**Anorexia**: an eating disorder that involves restricted feeding, leading to low body weight.

**Asthenia**: a symptom characterized by generalized weakness and/or lack of energy.

**Axenic flies**: flies without gut microbes.

**Binary system**: genetic tools for transgene expression and genetic manipulations. Three binary systems, GAL4/UAS, LexA/LexAop and QF/QUAS, have been established in *Drosophila* and termed according to their different components.

**Catabolic metabolism**: also termed catabolism; a group of metabolic pathways that breaks down molecules into smaller units.

**Ensheathing glia**: a subtype of glial cells that enwrap major structures in the brain.

**Eye imaginal disc**: a sac-like epithelial structure inside the larvae of insects, which gives rise to the adult eye.

**IGF-1/AKT pathway**: a conserved signaling pathway, regulated by insulin-like growth factor-1 (IGF-1), that activates the PI3K/AKT pathway by phosphorylating AKT and regulates cell proliferation, growth and survival.

**Indirect flight muscles**: power-producing muscles that move the wings indirectly by deforming the thoracic exoskeleton.

**Insulin resistance**: a condition whereby cells do not respond properly to insulin.

**Janus kinase/signal transducer and activator of transcription (JAK/STAT) signaling**: a conserved signaling pathway involved in processes including development, immunity and cancer.

**Lipid mobilization**: increased breakdown of lipids to release energy.

**Tumor necrosis factor alpha (TNF-α) pathway**: a conserved signaling pathway regulated by TNF-α that is involved in inflammatory responses and tumor necrosis.

Resistance to anabolic metabolism (Box 1) signals and an elevated rate of systemic catabolic metabolism (Box 1) are hallmarks of tumor-triggered host metabolic alterations, with well-documented dysregulation of carbohydrate, fat and protein metabolism. The altered carbohydrate metabolism in cancer patients has been recognized for over a century (Rohdenburg et al., 1919). Although cancer patients have normal fasting blood sugar levels, they manifest a decreased glucose utilization rate in intravenous glucose tolerance tests (Bishop and Marks, 1959). Because tumor cells actively absorb glucose, this decrease must be due to changes in other host organs, such as increased glucose production or a reduction in the rate of glucose utilization in peripheral organs. Either of these changes could lead to a decrease in body glycogen and fat storage (Holroyde et al., 1984; Mulligan et al., 1992). In addition, fat loss is thought to be caused not only by a reduction in glucose utilization and lipid synthesis but also by enhanced lipid mobilization (Box 1) (Das et al., 2011). In support of this hypothesis, mice that are deficient for adipose triglyceride lipase (Box 1) are protected from tumor-associated fat loss (Das et al., 2011). Cachexia patients also show abnormally high blood levels of free fatty acids, glycerol and triacylglycerol, which are indicative of elevated lipid mobilization (Das and Hoefler, 2013; Shaw and Wolfe, 1987). Tumors also lead to the dysregulation of protein and amino acid metabolism in muscles (Argilés et al., 2014). Because balanced protein synthesis and degradation is essential for muscle maintenance (Bonaldo and Sandri, 2013), a loss of this balance leads to muscle wasting. These and other findings highlight that carbohydrate, lipid and protein metabolism are among the targets of host metabolic alterations triggered by tumors.

Interestingly, these metabolic alterations occur in multiple organs, indicating that tumors can trigger changes in non-tumor tissues via the activity of tumor-secreted factors. Through the release of these factors, tumors can remotely affect other organs and can reprogram systemic metabolism (Argilés et al., 2019; Levental et al., 2009; Provenzano et al., 2006). Thus, to better understand the mechanisms underlying cachexia, we first need to identify the factors that emanate from tumors. Over the years, a number of proteins secreted from tumors have been identified and implicated in cancer cachexia, including growth factors, cytokines, interleukins and chemokines (Paltridge et al., 2013; Zhong et al., 2008). In particular, studies in mouse models and in cancer patients have shown that interleukin-6 and -1 (IL-6/IL-1) not only sustain tumor growth, survival and progression (Coussens and Werb, 2002), but also perturb host metabolic homeostasis, leading to cachexia (Baltgalvis et al., 2008; Graziano et al., 2005; Kuroda et al., 2007; Mantovani et al., 2010; Uehara et al., 1989). Another example is growth differentiation factor 15 (GDF15), which mediates tumor-induced body mass loss in xenograft mouse tumor models (Johnen et al., 2007; Lerner et al., 2016). In particular, a recent study has demonstrated that targeting the GDF15 receptor in the brain with a monoclonal antibody could reverse cancer cachexia phenotypes in mice (Suriben et al., 2020). Altogether, deciphering the physiological roles of known cachectic factors, as well as identifying new ones, would further our understanding of cancer cachexia.

*Drosophila* is a widely used model organism in biomedical research, especially as more than 75% of human disease-associated genes are conserved in the fly (Bier, 2005; Reiter et al., 2001). As a model organism that has been used for over a century, a wealth of genetic tools is available for *Drosophila* studies (Ugur et al., 2016). In recent years, *Drosophila* has emerged as a powerful model in which to identify tumor-derived factors that trigger systemic organ wasting (Bilder et al., 2021). These studies have identified novel organ wasting/cachexia factors and have provided insights into the molecular basis of the roles of these factors in tumor-induced metabolic dysregulation (Table 1). In this Review, we focus on the interaction between tumor and host metabolism and highlight recent advances in our understanding of organ wasting/cachexia from *Drosophila* studies.

**Table 1.**
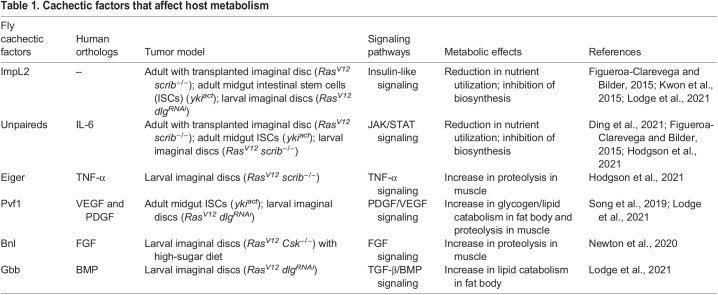
Cachectic factors that affect host metabolism

## *Drosophila* models of organ wasting induced by tumors

**Fig. 1.**
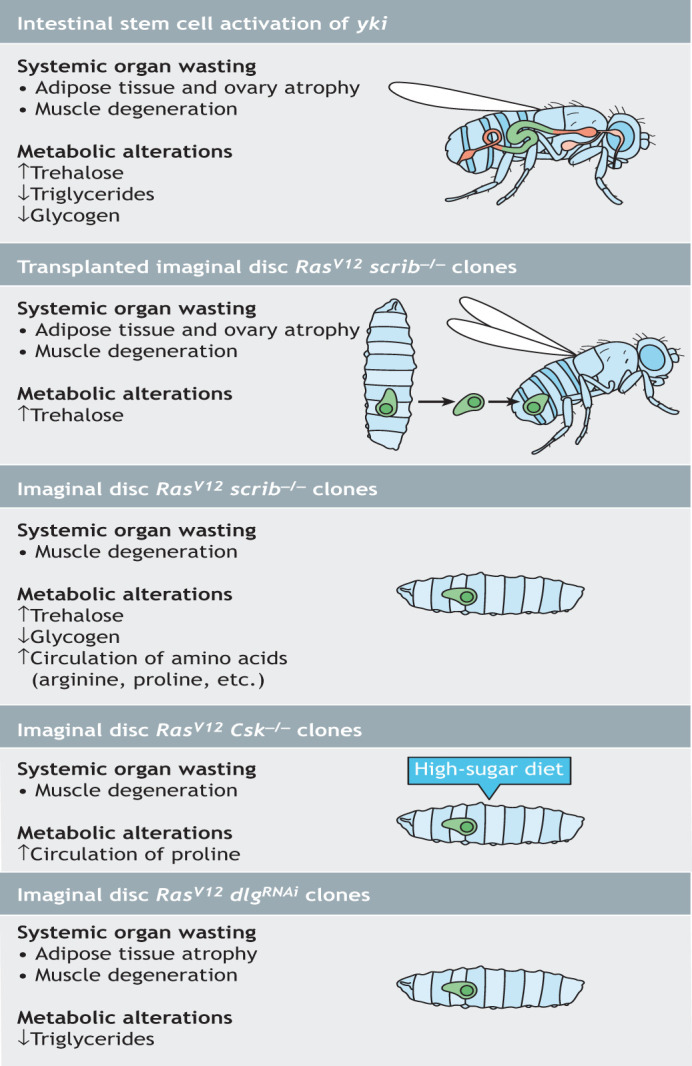
***Drosophila* tumor models associated with cachexia.** Schematics show *Drosophila* tumor models and their cachexia-related phenotypes of adult intestinal *yki^act^* tumor; adult imaginal disc *Ras^V12^ scrib^−/−^* tumor; larval imaginal disc *Ras^V12^ scrib^−/−^* tumor; larval imaginal disc *Ras^V12^ Csk^−/−^* tumor; and larval imaginal disc *Ras^V12^ dlg^RNAi^* tumor. *Csk*, *C-terminal Src kinase*; *dlg*, *disc-large 1*; *Ras^V12^*, a constitutively active form of *Ras oncogene at 85D*; *scrib*, *scribble*; *yki*, *yorkie*.

Several *Drosophila* tumor models have been used to study organ wasting (Fig. 1). Two different *Drosophila* tumor models with organ-wasting phenotypes have been generated in adult flies through activation of oncogenic pathways (Figueroa-Clarevega and Bilder, 2015; Kwon et al., 2015). One such model, *yki^act^*, is based on the overexpression of an active form of the transcriptional coactivator *Yap1* oncogene ortholog *yorkie* (*yki*), known to lead to aberrant cell proliferation (Oh and Irvine, 2009). Specifically, expression of *yki^act^* in adult intestinal stem cells generates gut tumors and systemic organ wasting (Fig. 1) (Kwon et al., 2015). The other model, *Ras^V12^ scrib*^−/−^, is based on the expression of oncogenic *Ras^V12^* in the *scribble* (*scrib*) tumor suppressor mutant background, a well-established system to induce malignant tumors in flies (Brumby and Richardson, 2003; Pagliarini and Xu, 2003). Transplantation of *Ras^V12^ scrib*^−/−^ tumorous eye imaginal discs (Box 1) into adult flies triggers organ wasting (Fig. 1) (Figueroa-Clarevega and Bilder, 2015). In both models, adult flies develop large tumors and a bloating phenotype, together with the degeneration of the fat body, muscle and ovaries, and Imaginal morphogenesis protein-late 2 (ImpL2) was identified as a tumor-derived cachectic factor. ImpL2 is a secreted insulin-binding protein that antagonizes insulin signaling (Honegger et al., 2008). Two additional factors were also identified in the *yki^act^* gut tumor model, PDGF- and VEGF-related factor 1 (Pvf1) and the IL6-like cytokine Unpaired 3 (Upd3), and shown to contribute to wasting (Song et al., 2019; Ding et al., 2021). However, it remains to be determined whether either of these factors play a role in organ wasting in the *Ras^V12^ scrib*^−/−^ tumor model. Interestingly, another Upd protein, Upd2, is secreted by *Ras^V12^ scrib*^−/−^ tumor cells. However, its role in the context of body wasting remains unclear (Figueroa-Clarevega and Bilder, 2015).

Additional models of organ wasting have been generated in *Drosophila* larvae. In the first, *Ras^V12^ scrib*^−/−^ clones were induced in eye imaginal discs (Fig. 1) (Hodgson et al., 2021). Interestingly, although tumor cells in this model are genetically identical to the adult *Ras^V12^ scrib*^−/−^ tumors described above, larval tumors employ a different mechanism to induce body wasting as they promote muscle breakdown by activating the janus kinase/signal transducer and activator of transcription (JAK/STAT; also known as Hop/Stat92E in fly) and tumor necrosis factor alpha (TNF-α; also known as Egr in fly) pathways (Box 1) (Hodgson et al., 2021). Further, a different study that used micro-computed X-ray tomography and carbon tracing, showed that *Ras^V12^ scrib*^−/−^ imaginal disc tumors increase their mass at the expense of larval muscle tissue, which undergoes wasting through increased autophagy (Khezri et al., 2021). Of note, the role of ImpL2, the determinant cachectic factor in the adult models (Table 1), was not explored in this larval model (Hodgson et al., 2021).

The second larval model, *Ras^V12^ Csk^−/−^*, was generated by expressing activated Ras in *Csk* mutant clones in eye imaginal discs (Fig. 1) (Hirabayashi et al., 2013). Deletion of C-terminal Src kinase (CSK) is known to cooperate with oncogenic Kras to stimulate pancreatic neoplasia in mice (Shields et al., 2011). Interestingly, this tumor model is diet dependent, as *Ras^V12^ Csk^−/−^* tumors grow preferentially in larvae fed a high-sugar diet (Hirabayashi et al., 2013). A recent report described *Ras^V12^ Csk^−/−^* larval tumors secreting the fibroblast growth factor (FGF) ligand *branchless* (*bnl*), which in turn induces muscle wasting (Newton et al., 2020). As in the *Ras^V12^ scrib*^−/−^ larval tumor model, whether the cachectic ImpL2 plays a role in this model is not clear.

A third larval cancer cachexia model, *Ras^V12^ dlg^RNAi^* (Lodge et al., 2021), was also generated in the eye-antennal epithelial discs by expressing activated Ras in a previously described genetic background in which the activity of the cell polarity tumor suppressor gene *disc-large 1* (*dlg*; also known as *dlg1*) (Woods and Bryant, 1991) is reduced (Fig. 1). These tumors were found to secrete two known cachexia factors, ImpL2 and Pvf1, as well as Matrix metalloproteinase 1 (Mmp1). Mmp1 downregulates fat body transforming growth factor beta (TGF-β) signaling, independently of ImpL2, by controlling the availability of one of its ligands, Glass bottom boat (Gbb). In addition, elevated *Mmp1* levels perturb fat body and muscle basement membrane (BM) and localization of extracellular matrix (ECM) proteins, leading to degeneration of the fat body and muscle (Lodge et al., 2021). Increased *Mmp1* expression has also been observed in *Ras^V12^ scrib*^−/−^ and *Ras^V12^ Csk^−/−^* larval tumor models, as well as in the adult *yki^act^* tumor model (Uhlirova and Bohmann, 2006; Hirabayashi et al., 2013; Song et al., 2019). Whether *Mmp1* expression is elevated in the transplanted imaginal disc *Ras^V12^ scrib*^−/−^ adult model remains unclear. Additionally, how Mmp1 and the other identified cachectic factors interact in these tumor models to trigger body wasting remains to be determined.

**Fig. 2.**
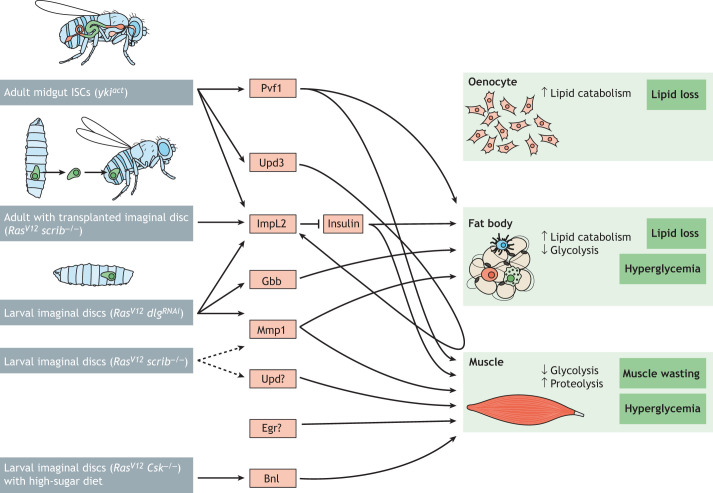
**Crosstalk between *Drosophila* tumors and host tissues.** A schematic showing *Drosophila* cachectic factors known to be secreted from adult and larval tumor models (genotypes shown in brackets) that are associated with pathologies in the oenocyte, fat body and muscle. Increased lipid catabolism in oenocytes and the fat body leads to lipid loss, while reduced glycolysis in the fat body and muscle causes hyperglycemia. Elevated proteolysis in the muscle leads to muscle wasting. Dashed lines indicate predicted regulatory interactions. Tumors in *Drosophila* tissues are shown in green. Bnl, Branchless; *Csk*, *C-terminal Src kinase*; *dlg*, *disc-large 1*; Egr, Eiger; Gbb, Glass bottom boat; ImpL2, Ecdysone-inducible gene L2; ISC, intestinal stem cell; Mmp1, Matrix metalloproteinase 1; Pvf1, PDGF- and VEGF-related factor 1; *Ras^V12^*, a constitutively active form of *Ras oncogene at 85D*; *scrib*, *scribble*; Upd, Unpaired; Upd3, Unpaired 3; *yki*, *yorkie*.

Each of the models described above has advantages and limitations. The presence of tumors in larvae is usually associated with delays in larval growth, which may confound interpretation of the tumors' effects on peripheral tissues (Hodgson et al., 2021; Khezri et al., 2021; Newton et al., 2020; Lodge et al., 2021). In addition, the time scale for larval models is much shorter than that for adult ones and may not be sufficient to fully evaluate the effect of tumors on the host. Regarding the adult tumor models, on one hand, tissue transplantation in adult flies is invasive and needs to be properly controlled to reliably generate comparable tumor-bearing animals. On the other hand, as transplantable tumor cells can be injected in animals of various genotypes, this approach can simplify the development of complex animal models in which to dissect tumor–host communication (Kim et al., 2021). Achieving similar goals in non-transplanted models requires two binary systems (Box 1) (del Valle Rodríguez et al., 2012), such as GAL4/UAS and LexA/LexAop (Lee et al., 2021) or GAL4/UAS and QF/QUAS (Hodgson et al., 2021; Lodge et al., 2021). Despite their limitations, research in fly models has identified a number of cachectic factors (Fig. 2) that we describe in detail below.

## *Drosophila* cachectic factors

### IGFBP/ImpL2

ImpL2 is secreted from the *yki^act^* and *Ras^V12^ scrib*^−/−^ adult tumor models and the larval *Ras^V12^ dlg^RNAi^* model associated with cachexia (Figueroa-Clarevega and Bilder, 2015; Kwon et al., 2015). In the hemolymph, ImpL2 can bind to *Drosophila* insulin-like peptides (dILPs; also known as Ilps) and counteract insulin/insulin-like growth factor (IGF) signaling (Honegger et al., 2008; Roed et al., 2018). Insulin/IGF signaling is an anabolic pathway that is essential for maintaining muscle homeostasis and for sustaining glycogen and lipid synthesis (Cahill et al., 1972; Saltiel and Kahn, 2001). Therefore, high hemolymph levels of ImpL2 reduce systemic insulin/IGF signaling, which inhibits glycogen synthesis and lipogenesis, ultimately leading to organ wasting. The fly studies suggest that insulin resistance caused by ImpL2 may play a role in cancer cachexia. Indeed, insulin resistance has been observed in several animal models of cancer cachexia and in cancer patients, indicating that the dysregulation of insulin signaling might be connected to cancer cachexia (Asp et al., 2010; el Razi Neto et al., 1996; Fearon et al., 2012; Fernandes et al., 1990; Guaitani et al., 1982; Lazarus et al., 1999; Tanaka et al., 1990). Mammalian IGF-binding proteins (IGFBP) are functionally similar to ImpL2, as both proteins antagonize insulin/IGF signaling (Baxter, 2014). A recent study of pancreatic ductal adenocarcinoma patients links mammalian IGFBP2 to cachexia (Dong et al., 2021), suggesting that the insulin/IGF signaling-related mechanism of cancer cachexia is conserved. Interestingly, while both ImpL2 and human IGFBPs antagonize the binding of human IGF-1, their structures and their binding to IGF-1 differ and involve distinct binding mechanisms, which may provide novel translational opportunities (Roed et al., 2018).

### Interleukin/Unpaired

Three Unpaired (Upd) genes – *Upd1*, *Upd2* and *Upd3* – are present in *Drosophila*. They encode related ligands that activate the JAK signaling pathway following binding to a gp130-like receptor, Domeless (Dome). Activation of JAK/Hop in turn phosphorylates the STAT/Stat92E transcription factor (Gilbert et al., 2005; Harrison et al., 1998) that dimerizes and translocates to the nucleus, where it promotes the expression of genes that regulate growth, development and metabolism (Amoyel et al., 2014; Herrera and Bach, 2019). Interestingly, the expression levels of two Upd genes are increased in *Drosophila* tumors. *Upd2* expression is elevated in the imaginal disc *Ras^V12^ scrib*^−/−^ tumor model, although no evidence has been found that it stimulates wasting (Figueroa-Clarevega and Bilder, 2015). *Upd3* expression is induced in the midgut *yki^act^* tumor model, and it not only sustains tumorigenesis but also leads to muscle wasting and fat loss (Ding et al., 2021). JAK/STAT signaling ligands have a similar role in cancer cachexia patients. Upd3 is a homolog of human IL-6, which drives systemic inflammation and is often associated with cancer cachexia (Fearon et al., 2012; Jung and Choi, 2014; Petruzzelli and Wagner, 2016; Smitka and Marešová, 2015). Like Upd3, IL-6 activates JAK/STAT signaling in host organs and leads to weight loss (Belizário et al., 2016; Bonetto et al., 2012; Ma et al., 2017; Miller et al., 2017), indicating a conserved role for IL-6/Upd3 in cancer cachexia. Of note, the activation of JAK/STAT signaling in *Drosophila* peripheral organs upregulates ImpL2 expression, thus exacerbating insulin resistance and contributing to the body-wasting phenotype (Ding et al., 2021), although it is currently not clear whether JAK/STAT pathway activation in cancer patients contributes to insulin resistance.

### TNF-α/Eiger

*eiger* (*egr*) is the single *Drosophila* ortholog of human TNF-α (also known as TNF) (Igaki et al., 2002; Moreno et al., 2002). In the *Ras^V12^ scrib*^−/−^ larval model, expression of the TNF-α/Egr receptor Wengen (Wgn) is upregulated in muscles. Knockdown of *wgn* in all muscles attenuates muscle degeneration in tumor-hosting larvae, indicating a role for TNF-α/Egr signaling in muscle wasting (Hodgson et al., 2021). Although the source of Egr in this model has not clearly been identified, previous studies have demonstrated that *egr* expression can be induced in tumors and in tumor-associated immune cells (Cordero et al., 2010; Igaki et al., 2009), suggesting that *egr* is upregulated due to the larval *Ras^V12^ scrib*^−/−^ tumor. TNF-α/Egr signaling is conserved between fly and humans. Human TNF-α, also termed cachectin, was initially believed to be critical in cancer cachexia (Beutler and Cerami, 1986; Oliff et al., 1987). A few studies in cell lines and animal models reported that TNF-α stimulates proteolysis and may cause the loss of protein from the body, leading to muscle degeneration (Han et al., 1999; Patel and Patel, 2017). However, older studies found that, although TNF-α can induce many systemic effects, it did not affect muscle loss (Kettelhut and Goldberg, 1988; Kettelhut et al., 1987). Further, targeting TNF-α with neutralizing antibodies did not prevent body wasting in cancer patients (Jatoi et al., 2010). Thus, the role of TNF-α in cancer cachexia requires further exploration. Interestingly, TNF-α can induce insulin resistance in mice (Hotamisligil, 1999), and a recent study in flies observed that fat body-secreted Egr targets insulin-producing cells to inhibit the expression of dILPs (Agrawal et al., 2016), indicating a conserved role for TNF-α/Egr signaling in antagonizing insulin/IGF signaling.

### PDGF/VEGF/Pvr signaling

*Drosophila* Pvf1 stimulates body wasting in the adult *yki^act^* tumor model (Song et al., 2019). Tumor-secreted Pvf1 binds to its PDGF- and VEGF-receptor related (Pvr) receptor in host fly organs, including in muscles and the fat body, to activate downstream MEK (also known as Dsor1 in fly) signaling. MEK activation in turn enhances levels of catabolism and host wasting independently of ImpL2 expression, although the targets of MEK signaling remain unclear (Song et al., 2019). MEK activation by VEGF is also observed in humans. Activated VEGF signaling leads to phosphorylation of protein kinase C (PKC; also known as PRKC) which in turns stimulates the Raf/MEK/ERK (also known as RAF1/MAP2K/MAPK) pathway (Beamer et al., 2010; Nilsson and Heymach, 2006). Human VEGF and PDGF facilitate tumor growth by inducing angiogenesis (Cao, 2013; Carmeliet, 2005; Chang et al., 2000), which enhances the supply of nutrients and oxygen to tumor cells (Parmar and Apte, 2021). However, whether VEGF and PDGF signaling in humans also targets host organs to stimulate body wasting in cancer patients requires further study.

### FGF/Branchless

The Fibroblast growth factor (FGF) ortholog *bnl* is a *Drosophila* FGF family ligand secreted by the imaginal disc *Csk^−/−^ Ras^V12^* larval tumor under high-sugar-diet conditions. Bnl is necessary and sufficient to promote tumor growth while inducing muscle degradation (Newton et al., 2020). Although our current understanding of the role of human FGF family ligands in cancer cachexia remains incomplete, several of the 18 human FGF family members have been implicated in systemic metabolic regulation (Ornitz and Itoh, 2015). In particular, FGF21 is a known regulator of energy metabolism, and high serum levels of FGF21 have been found in age-related cachexia patients (Franz et al., 2019), suggesting a potential role for FGF21 in cancer cachexia. However, further investigation is necessary to support this hypothesis.

### BMP/Gbb

The *Drosophila gbb* gene encodes a Bone morphogenetic protein (BMP) ligand of the transforming growth factor beta (TGF-β) signaling pathway. Gbb was identified in the *Ras^V12^ dlg^RNAi^* larval imaginal disc tumor model (Lodge et al., 2021). Elevated levels of *Mmp1* in tumor cells stimulate *gbb* expression and secretion. Tumor-derived Gbb in turn activates TGF-β/BMP signaling in both host fat body and muscle, leading to organ wasting (Lodge et al., 2021) by a mechanism that remains to be elucidated. Interestingly, reduced BMP signaling is a known cause of muscle wasting in cancer cachexia patients and in the transplantable colon 26 (C-26) adenocarcinoma mouse model (Sartori et al., 2021). These studies suggest that proper levels of TGF-β/BMP signaling are important for muscle maintenance, and that tumor-induced alterations in TGF-β/BMP signaling may contribute to cancer cachexia.

## Metabolite signatures of cachexia

Abnormal metabolite levels are frequently observed in tumors, and some metabolites promote tumor growth by altering metabolic flux (Sullivan et al., 2016). Some changes in metabolites are specific to cachexia in cancer patients, including reduced serum levels of fatty acids, leucine and valine, as well as elevated serum levels of glucose, lysine, methionine, glutamate, succinate, pyruvate and alanine (Yang et al., 2018). Similarly, a study of a murine model of cancer cachexia reported cachexia-related changes in metabolites, including amino acids, carbohydrates and lipids (Der-Torossian et al., 2013), although minor differences exist that probably reflect differences in tumor types. Identifying the metabolite signatures of cancer cachexia could provide insights into cancer development and diagnosis.

**Fig. 3.**
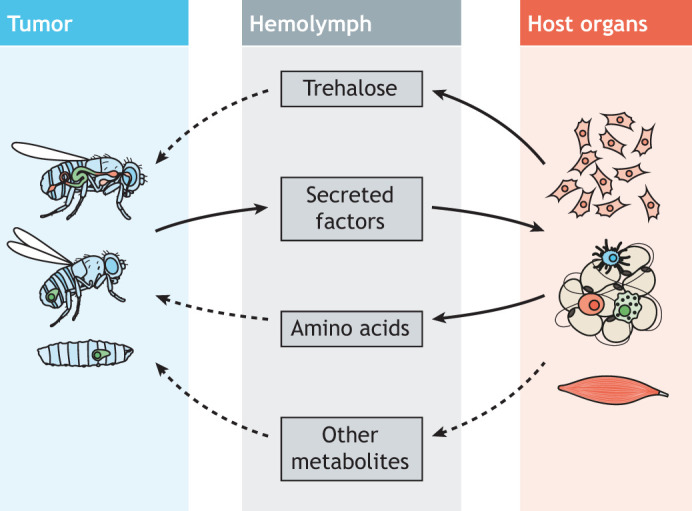
**Metabolite changes in *Drosophila* tumor models.** Tumor-secreted factors stimulate host organs to release metabolites into the hemolymph. Metabolites that are typically elevated in *Drosophila* tumor models include trehalose and amino acids. Tumors in *Drosophila* tissues are shown in green. Dashed lines indicate predicted directions.

Abnormal levels of metabolites in cancer patients are caused by tumor-driven metabolic reprogramming at the whole-organism level, which enhances the release of energy and nutrients from body storage and redirects the energy flux to fulfill the demands of cancer cell hyperproliferation (Baracos et al., 2018; Pavlova and Thompson, 2016; Sullivan et al., 2016). Indeed, a few circulating metabolites are required for the development of certain tumors, including lipids (Lewis et al., 2015; Nieman et al., 2011), branched amino acids (Tönjes et al., 2013), glutamine (Dang, 2012; Son et al., 2013), serine (Maddocks et al., 2013) and glucose (Duan et al., 2014; Sharma et al., 2018). Cancer cells actively take up these nutrients, gaining an advantage that leads to the resistance of cancer cells to nutrient deprivation and that fuels their proliferation (Kamphorst et al., 2015). *Drosophila* tumor models also display abnormal levels of metabolites (Fig. 3). Increased trehalose, the ‘blood sugar’ of *Drosophila*, has been observed in both larval and adult *Drosophila* tumor models (Figueroa-Clarevega and Bilder, 2015; Kwon et al., 2015; Khezri et al., 2021). In the adult *yki^act^* and *Ras^V12^ scrib*^−/−^ tumor models, this hyperglycemia is largely due to the systemic reduction of insulin signaling by tumor-derived ImpL2 (Figueroa-Clarevega and Bilder, 2015; Kwon et al., 2015), whereas in the *Ras^V12^ scrib*^−/−^ larval tumor model, it is a result of systemic autophagy (Khezri et al., 2021). Interestingly, elevated autophagy in this model also increased serum levels of amino acids, including arginine, proline, glutamine, serine, alanine and lysine. In a different *Drosophila* obesity-enhanced tumor model, proline is released through muscle degradation, which promotes tumor growth (Newton et al., 2020). The role of other metabolites in these fly tumor models, such as branched amino acids and circulating lipids, remains to be characterized. Fly models of different oncogene mutations also have distinct metabolic properties, even when generated in the same organ (Gándara et al., 2019). Together, these findings indicate that *Drosophila* tumor models may recapitulate the metabolite signatures observed in cancer patients, thus providing a platform to explore systemic metabolic changes induced by tumor–host organ crosstalk.

## Adipose tissue/fat body wasting in the context of cachexia

An outcome of cachexia is a significant decrease in body fat, which is associated with disease progression (Fouladiun et al., 2005; Prado et al., 2013). This is not due to a loss of fat cells but rather to increased systemic lipolysis (Hall and Baracos, 2008; Rydén and Arner, 2007; Zuijdgeest-van Leeuwen et al., 2000). Tumor-secreted factors, such as IL-6 (Han et al., 2018) and TNF-α (Patel and Patel, 2017), have been proposed to inhibit lipogenesis and/or to promote lipolysis. The insulin resistance that is associated with cancer cachexia is another possible mechanism that could account for fat loss, as insulin signaling suppresses lipolysis through mTORC1 (Cignarelli et al., 2019; Dong et al., 2021; Lindh et al., 2007). Consistent with this, in the adult fly cachexia models, ImpL2 leads to adipose tissue wasting (Figueroa-Clarevega and Bilder, 2015; Kwon et al., 2015), reminiscent of the recent finding that IGFBP2 is a biomarker for cancer cachexia in human pancreatic ductal adenocarcinoma patients (Dong et al., 2021). Interestingly, RNA interference (RNAi)-mediated knockdown of *ImpL2* in the *yki^act^* tumor model partially rescues body-wasting phenotypes, indicating the existence of additional factor(s) involved in adipose tissue wasting (Kwon et al., 2015). One candidate is Pvf1, which is secreted from *Drosophila* intestinal *yki^act^* tumor cells. Pvf1 activates MEK/ERK signaling, and elevated MEK signaling in adipose tissue impairs lipolysis and lipid storage (Song et al., 2019). In addition, a single-cell survey of *Drosophila* abdominal tissues has recently revealed that Pvr, the Pvf1 receptor, is enriched in the hepatocyte-like cells/oenocytes (Ghosh et al., 2020). Because muscle-derived Pvf1 stimulates Pi3K/Akt/Tor signaling in oenocytes and mobilizes lipid storage (Ghosh et al., 2020), it is possible that *yki^act^* tumor-secreted Pvf1 induces lipid loss through the same mechanism. In addition, Gbb, identified in the *Ras^V12^ dlg^RNAi^* larval tumor model, may also be involved in fat body wasting (Lodge et al., 2021). In this model, Gbb is secreted from tumor cells and activates Gbb/BMP/TGF-β signaling in the fat body, which leads to increased lipid mobilization. Mmp1 is secreted together with Gbb from the tumor, and both Mmp1 and Gbb can disrupt the BM/ECM of the fat body, which might contribute to increased lipid mobilization (Lodge et al., 2021). However, whether lipid metabolism is directly affected by Gbb/BMP/TGF-β signaling or is an outcome of BM/ECM disruption requires further investigation. Together, these studies suggest that fat atrophy in cancer patients probably results from the combined effects of a variety of tumor-derived factors.

## Muscle wasting in the context of cachexia

In advanced stages of cancer, the most obvious manifestation of cachexia is muscle wasting, a significant reduction in skeletal muscle mass driven by increased proteolysis, which leads to subsequent weight loss (Tisdale, 2009; Argilés et al., 2014; Fearon et al., 2012; Petruzzelli and Wagner, 2016). Different types of cancers induce cachexia to varying extents, with pancreatic cancers having the highest association with weight loss (Baracos et al., 2018). One pathway that positively regulates muscle mass in mice is the IGF-1/PI3K/AKT pathway (Lai et al., 2004). In the C-26 mouse model of cachexia, lower levels of phosphorylated AKT in muscle correlate with muscle wasting (Asp et al., 2010) and with increased expression in muscle of two key muscle-specific ubiquitin E3 ligases of the ubiquitin–proteasome system (Asp et al., 2010; Talbert et al., 2019): muscle ring finger-1 (MuRF1; also known as TRIM63) and muscle atrophy F-box (MAFbx; also known as FBXO32)/atrogin-1 (Bodine et al., 2001; Gomes et al., 2001). The autophagy–lysosome pathway also promotes protein degradation and removal of dysfunctional mitochondria, a process called mitophagy (He and Klionsky, 2009). Under increased rates of autophagic flux, double-membrane vesicles called autophagosomes capture protein aggregates and defective mitochondria and then fuse to lysosomes, where hydrolases promote proteolysis (He and Klionsky, 2009). Forced expression of a constitutively active form of FoxO3, a transcription factor negatively regulated by the IGF-1/AKT pathway (Box 1), in isolated mouse muscle fibers or in C2C12-derived myotubes increases proteolysis and atrophy while upregulating the expression of *Bnip3*, an inducer of autophagy and mitophagy (Mammucari et al., 2007; Zhao et al., 2007). Further, cachexic muscles of the C-26 mouse model displayed increased expression of *Bnip3* (Asp et al., 2010; Talbert et al., 2019; Cornwell et al., 2014) and higher autophagic flux (Penna et al., 2013), suggesting that the autophagy–lysosome pathway might also play a role in muscle wasting during cancer-induced cachexia. Altogether, research in animal models of cachexia highlights a potential link between lower IGF-1/AKT signaling in muscle and increased proteolysis during cachexia. However, evidence from cancer patients with cachexia has been less clear in showing increased expression of MuRF1, MAFbx and *BNIP3* in muscle (Talbert et al., 2019; Gallagher et al., 2012; Bonetto et al., 2013; D'Orlando et al., 2014), arguing for additional models and mechanisms of cancer-induced muscle wasting.

In the two fly tumor models that produce *ImpL2*, insulin signaling is reduced in peripheral tissues and systemic organ wasting is observed (Figueroa-Clarevega and Bilder, 2015; Kwon et al., 2015). In both models, the thoracic muscles show severely degenerated myofibers, which is associated with impaired muscle function. It is currently not known whether an increase in ubiquitin–proteasome system activity or higher levels of autophagy lead to muscle degeneration (Figueroa-Clarevega and Bilder, 2015; Kwon et al., 2015). Nevertheless, the mitochondria of the indirect flight muscles (Box 1) exhibit abnormal morphology and the thoraces of flies with tumors have lower ATP content, which indicate changes in energy metabolism and mitochondrial dysfunction (Figueroa-Clarevega and Bilder, 2015; Kwon et al., 2015). Specific RNAi-mediated knockdown of *ImpL2* in the adult *yki^act^* gut tumors or in the *Ras^V12^ scrib*^−/−^ adult tumor models restored insulin signaling in peripheral tissues and ameliorated the muscle phenotypes without affecting tumor growth (Figueroa-Clarevega and Bilder, 2015; Kwon et al., 2015), demonstrating that a tumor-derived factor can disrupt insulin signaling and drive organ wasting (Wagner and Petruzzelli, 2015). Interestingly, increasing the activity of the Wnt/Wingless signaling pathway specifically in the muscles of flies with gut *yki* tumors had a protective role by increasing insulin signaling and counteracting the effect of ImpL2 in muscle wasting. These findings suggest that Wnt signaling might be of therapeutic value in the treatment of cachexia, especially muscle wasting (Lee et al., 2021).

Given the heterogeneous nature of cancer-induced cachexia, it is possible that muscle wasting is driven by a combination of factors secreted by the same tumor (Freire et al., 2020). In *Drosophila*, transcriptomic analysis of gut *yki* tumors or larval wing imaginal discs with mutant *scrib* or *dlg* have shown that several secreted factors are upregulated (Figueroa-Clarevega and Bilder, 2015; Song et al., 2019). Studies using the gut *yki-*tumor model identified two other factors derived from gut tumors in addition to ImpL2, Pvf1 and Upd3, which can impair muscle function and induce proteolysis in flight muscles (Ding et al., 2021; Song et al., 2019). In addition, a recent study of a larval neoplastic epithelial tumor (*Ras^V12^ dlg^RNAi^*) model proposed that tumor-secreted Mmp1 contributes to host muscle wasting by disrupting the BM/ECM of muscle (Lodge et al., 2021). Interestingly, ImpL2 produced by these tumors acts in parallel to Mmp1 by increasing autophagy and reducing protein synthesis in muscles (Lodge et al., 2021). Taken together, even though most studies on the mechanisms of cancer-induced cachexia and muscle wasting have relied on mammalian models with transplanted tumors, recent studies in *Drosophila* have revealed similarities between flies and cancer patients, particularly at the level of systemic insulin signaling changes modulated by tumor-derived ImpL2 (Figueroa-Clarevega and Bilder, 2015; Kwon et al., 2015; Lodge et al., 2021) or IGFBP2 in cancer patients (Dong et al., 2021). Thus, *Drosophila* studies can provide an additional platform to identify conserved genes directly involved in muscle degradation/wasting or new tumor-derived factors that can promote systemic organ wasting.

## Future perspectives and outstanding questions

*Drosophila* studies of tumor models associated with organ wasting have led to many insights into tumor-secreted factors and their roles in organ wasting and metabolism alteration. In particular, characterization of factors derived from different fly tumor cachexia models highlight that both common and distinct cachectic molecules are produced by different tumor types (Fig. 2). These studies support the view that cachexia is more than one disease. Below, we highlight a number of outstanding questions that can be addressed using the fly cachexia models.

### Expanding the repertoire of tumor models

The fly studies discussed here underscore the need to study tumor models of different genotypes, at different developmental stages and under different diets, to fully characterize the various mechanisms underlying cachexia. Fortunately, inducing tumors in *Drosophila* is not challenging as, in most cases, they can be induced by manipulating one oncogene (Bilder et al., 2021), and a number of models are already available (Rudrapatna et al., 2012; Gonzalez, 2013). Whether cachexia occurs in these tumor models or not remains to be addressed. Another strategy to identify novel cachectic factors is to test different culture conditions. For example, *bnl* was identified in the *Ras^V12^ Csk^−/−^* tumor model when larvae were fed a high-sugar diet (Newton et al., 2020). Thus, we expect that the exploration of different tumor models and conditions will help identify new cachectic factors and elucidate their roles in tumor-induced organ wasting and metabolic disruption.

### Testing cachectic and non-cachectic models

Because not all *Drosophila* tumor models are associated with organ wasting, a detailed comparison of factors secreted by tumors associated with or without organ wasting may help evaluate the cachectic properties of a specific factor or combinations of factors. Indeed, a recent study examined *ImpL2* expression levels in cachectic and non-cachectic midgut intestinal stem cell tumor models and found that ImpL2 expression is highly increased in the cachectic *Ras^V12^ Notch^DN^* tumors but not in non-cachectic *Raf^gof^* overexpression tumors (Lee et al., 2021). Such comparative studies might help prioritize factors that are most likely to be relevant to cachexia.

### Identifying additional factors from tumors involved in cachexia

Previous studies identifying tumor-secreted factors in *Drosophila* have relied on genetic screens and bulk RNA sequencing. In a recent study, single-cell transcriptome analysis (scRNAseq) of the entire fly led to the annotation of all cell types in the adult (Li et al., 2022). Thus, full-body scRNAseq of tumor-model flies should facilitate and expedite the discovery of secreted cachectic factors both from the tumor itself and from peripheral tissues. In addition, proteomic methods such as proximity labeling of secreted proteins using engineered biotin ligase-based assays (Droujinine et al., 2021) could identify proteins trafficking between tumors and peripheral organs.

Fly studies have highlighted that both common and distinct cachectic molecules are produced by different tumor types (Fig. 2). For instance, ImpL2 is commonly produced by tumors in two adult fly tumor models, but the production of Pvf1 has only been reported in the gut intestinal stem cell tumor model (Figueroa-Clarevega and Bilder, 2015; Kwon et al., 2015; Song et al., 2019). Elucidating the genetic landscape of tumors that trigger the expression of specific factors will be required to understand the basis for such differences.

### System-level understanding of cachectic factors

Tumor-secreted factors activate signaling pathways in peripheral tissues. Thus, information about the expression levels of signaling pathway receptors and their target genes in peripheral organs may help identify which and where pathways are activated. Two resources described recently will facilitate such studies: the single-cell atlas of the adult fly (Li et al., 2022), which can be visualized and analyzed through https://flycellatlas.org; and FlyPhoneDB, a web-based resource of cell–cell communication predictions (Liu et al., 2022), with which potential ligand-pathway connections between tissues can be explored systematically.

### Identifying secreted factors from peripheral tissues

To date, most *Drosophila* tumor model studies have focused on the influence of tumors on peripheral tissues; however, non-tumor tissues in the host might also release signaling molecules in response to tumors that feed back to tumor cells or other organs. Understanding the role of peripheral organs in the organismal phenotype is an important and understudied question. Inter-organ communication is critical for maintaining systemic metabolic homeostasis (Castillo-Armengol et al., 2019; Liu and Jin, 2017). Thus, tumors might reprogram host metabolism indirectly by changing host organ–organ communication. Investigating changes in the expression of factors secreted from peripheral tissues using scRNAseq and proximity labeling approaches might help address this important question.

### Role of microbiota in cachexia

Cancers, especially intestinal cancers, alter the gut microbiota (Wang et al., 2012); thus, the role of microbiota in cancer cachexia deserves special attention. Although our current understanding of the crosstalk between microbiota and cancer cachexia remains incomplete, various studies have highlighted the importance of the gut microbiota in systemic metabolic homeostasis (Lau and Wong, 2018). For example, certain strains of bacteria prevent infection-induced wasting, which interestingly occurs via sustained IGF-1/PI3K/AKT pathway activity in murine muscles, indicating that the gut microbiota can remotely affect other host organ(s) (Palaferri Schieber et al., 2015). Consistent with this idea, introduction of certain gut bacteria strains in a mouse model of acute leukemia can antagonize muscle wasting through suppression of proinflammatory cytokines (Bindels et al., 2016, 2012). *Drosophila* is an established model for studying gut microbiome interactions. Generation and maintenance of germ-free/axenic flies (Box 1) is inexpensive and not technically demanding, and a diet-provided microbial inoculum can easily be used to re-conventionalize axenic flies (Broderick and Lemaitre, 2012; Koyle et al., 2016; Ren et al., 2007). Thus, exploring the role of the microbiota in influencing the wasting phenotype associated with various types of fly tumors may provide new insights into the interplay between the microbiota and host metabolism in the context of cancer cachexia.

### Feeding and cachexia

Anorexia (loss of appetite) is a known symptom of cancer that may contribute to body wasting. The current understanding of the etiology of anorexia remains insufficient, as few factors have been identified (Laviano et al., 2008). One of the known anorexia factors is leptin, a hormone produced by adipose cells and enterocytes. Cachectic gastric cancer patients have increased levels of circulating leptin compared to non-cachectic gastric cancer patients, suggesting a role for leptin in cachexia (Kerem et al., 2008). Leptin acts on the hypothalamus to mediate eating, and higher serum leptin levels reduce appetite (Bastard et al., 2006). In addition, food consumption may be affected by systemic inflammation (Wigmore et al., 1997), indicating a possible influence of proinflammatory cytokines on normal eating. In fact, proinflammatory signaling from tumors could act on the hypothalamus, leading to cancer anorexia (Grossberg et al., 2010). Many animal models are used to study the mechanisms of feeding behavior, including *Drosophila* (DeBoer, 2009; Siegfried et al., 2003). Indeed, a *Drosophila* cancer anorexia model was developed recently and used to identify how *yki^act^* tumors in the adult fly eye affect feeding behavior (Yeom et al., 2021). This study identified Dilp8, the ortholog of human insulin-like 3 peptide (INSL3), as a factor released from eye tumors that affects the expression of feeding neuropeptides in the brain. Strikingly, this mechanism appears to be conserved, as serum INSL3 levels are associated with cancer anorexia severity in patients with pancreatic cancer (Yeom et al., 2021). Of note, a recent *Drosophila* study showed that enteric infection-induced Upd2 and Upd3 secretion by the gut can activate JAK/STAT signaling in ensheathing glia (Box 1). This in turn perturbs olfactory discrimination and influences feeding behavior (Cai et al., 2021). Given that Upd3 expression is increased in the *yki^act^* gut tumor model (Ding et al., 2020; Song et al., 2019), and that Upd2, the fly equivalent of leptin (Rajan and Perrimon, 2012), is among the strongly elevated secreted factors in the Ras-driven tumor model (Figueroa-Clarevega and Bilder, 2015), it is possible that the olfactory sensory system could be affected in both models. Whether feeding behaviors are affected in various *Drosophila* tumor models and how this translates to patients remain to be thoroughly investigated.

## Conclusions

In summary, *Drosophila* research has contributed to our knowledge of secreted factors involved in cancer cachexia. Expanding our studies of *Drosophila* tumor models to identify new tumor-secreted factors relevant to cachexia, identifying the signaling pathways they affect and characterizing the metabolic outcomes of their activities, could improve our understanding of cancer cachexia and potentially lead to translational applications.
